# Effect of dual antiplatelet therapy prolongation in acute coronary syndrome patients with both high ischemic and bleeding risk: insight from the OPT-CAD study

**DOI:** 10.3389/fcvm.2023.1201091

**Published:** 2023-09-08

**Authors:** Kun Na, Miaohan Qiu, Ningxin Wei, Jiayin Li, Chenghui Yan, Jing Li, Yi Li, Yaling Han

**Affiliations:** ^1^ Department of Cardiology, Cardiovascular Research Institute, General Hospital of Northern Theater Command, Shenyang, China; ^2^ College of Life Sciences and Biopharmacy, Shenyang Pharmaceutical University, Shenyang, China; ^3^ College of Life Sciences and Health, Northeastern University, Shenyang, China; ^4^ Department of Cardiology, The Second Affiliated Hospital of Harbin Medical University, Harbin, China

**Keywords:** acute coronary syndrome, dual antiplatelet therapy, ischemic complications, bleeding complications, prognosis

## Abstract

**Background:**

In current clinical practice, controversy remains regarding the clinical benefits of prolonged dual antiplatelet therapy (DAPT) in acute coronary syndrome (ACS) patients facing high risks of both ischemia and bleeding (“bi-risk”) following percutaneous coronary intervention (PCI). This study aimed to investigate the feasibility of identifying a group of bi-risk ACS patients after PCI using the OPT-BIRISK criteria, emphasizing extended DAPT treatment safety and efficacy beyond 12 months in these bi-risk ACS after PCI in real-world conditions.

**Methods:**

This analysis compared extended DAPT and single antiplatelet therapy (SAPT) at 12–24 months in ACS patients undergoing PCI complicated with both ischemic and bleeding risk as defined by OPT-BIRISK criteria without premature DAPT discontinuation before 9 months or major clinical adverse events within 12 months. This was a *post hoc* analysis of the Optimal antiPlatelet Antiplatelet Therapy for Chinese Patients with Coronary Artery Disease (OPT–CAD) study. The main research outcome was the incidence of ischemic events within 12–24 months, which was determined as a composite of stroke, myocardial infarction, and cardiac death events. Through propensity score matching (PSM), groups were balanced. For the external validation of the OPT-BIRISK criteria to identify a bi-risk ACS patient, ischemic events, BARC 2, 3, 5 bleeding events, and BARC 3, 5 bleeding events at 5 years were analyzed.

**Results:**

The total number of ACS patients analyzed in this analysis was 7,049, of whom 4,146 (58.8%) were bi-risk patients and 2,903 (41.2%) were not. The frequency of ischemic events was significantly different between the two groups at 5 years (11.70% vs. 5.55%, *P* < 0.001), and the incidence of BARC 2,3,5 bleeding was significantly higher in the bi-risk group (6.90% vs. 4.03%, *P* < 0.001) than in the non-bi-risk group. Among the bi-risk patients without any clinical adverse events within 12 months that underwent extended DAPT treatment (*n* = 2,374, 75.7%) exhibited a lower risk of stroke at 12–24 months (1.10% vs. 2.10%, *P* = 0.036) relative to those that underwent SAPT (*n* = 763, 24.3%), while bleeding risk did not differ significantly between these groups. PSM cohort analysis results were consistent with those of overall group analyses.

**Conclusion:**

In conclusion, the findings showed that using the OPT-BIRISK criteria could help physicians identify ACS patients at a high risk of developing recurrent ischemia and bleeding episodes after PCI. Compared to antiplatelet monotherapy, a strategy of extended DAPT may offer potential benefits in lowering the risk of stroke without carrying a disproportionately high risk of serious bleeding problems among these patients who were event-free after a year of DAPT.

## 1. Introduction

Following the placement of a drug-eluting stent (DES), antiplatelet therapy is critical for secondary prevention in patients with acute coronary syndrome (ACS) (1). Balancing bleeding and ischemic risks is key to maximizing the benefits of antiplatelet therapy for patients. After DES implantations, it is currently advised in clinical practice that ACS patients receive dual antiplatelet treatment (DAPT) for at least 12 months, with the possibility of an additional 30 months if necessary (1). Data from the EPICOR-Asia trial indicated that a significant proportion of patients underwent prolonged DAPT treatment beyond 12 months to lower their risk of ischemia (2). Meanwhile, extended DAPT treatment does result in an elevated bleeding risk (3). However, An estimated 40% of ACS patients face high risks of both ischemia and bleeding events, and these individuals have been said to comprise a “bi-risk” ACS population (4). Accordingly, the optimal DAPT treatment duration for these bi-risk patients remains uncertain (5, 6).

This study was a *post hoc* analysis of the prospective Optimal Antiplatelet Therapy for Chinese patients with Coronary Artery Disease (OPT-CAD, NCT01735305) registry (7), designed to explore the feasibility of identifying a population of ACS patients facing both high risks of ischemia and bleeding (bi-risk) following PCI based on the OPT-BIRISK (4) criteria, with an additional focus on extended dual antiplatelet therapy (DAPT) treatment safety and efficacy beyond 12 months in these bi-risk ACS patients after receiving standard 9–12 months of DAPT without experiencing any adverse events under real-world conditions.

## 2. Methods

### 2.1. Data sources and study population

The study population was derived from the Optimal Antiplatelet Therapy for Chinese patients with Coronary Artery Disease (OPT-CAD) registry study (NCT01735305), a multicenter, prospective, observational study (7). Details of the study design have been previously published (7). The ethics committees of all participating centers approved the study, which was consistent with the Declaration of Helsinki. Participating patients provided written informed consent.

The present study was a *post hoc* analysis of ACS patients from the OPT-CAD trial that underwent at least one DES implantation. The inclusion criteria were as follows: (1) ACS patients undergoing DES implantation and complying with the bi-risk criteria (see the section Risk assessment); (2) receiving standard 9–12 months of DAPT without experiencing any adverse events, including myocardial infarction (MI), stroke, all-cause mortality, clinically driven revascularization, BARC type 2,3,5 bleeding, or in-stent thrombosis) within 12 months.

According to the DAPT treatment at 12–24 months, bi-risk patients were divided into prolonged DAPT or single antiplatelet therapy (SAPT) groups.

### 2.2. Risk assessment

OPT-BIRISK evaluation criteria (4): Patients <65 years old must meet at least 1 of the clinical criteria of high bleeding risk and at least 1 of the clinical criteria of high ischemic risk; Patients 65–75 years old must meet 1 of the clinical criteria of either high bleeding risk or high ischemic risk. OPT-BIRISK ischemic criteria were as follows: ≥75 years of age, multivessel coronary artery disease, target lesion requiring total stent length >30 mm, bifurcation lesions of Medina grade 0, 1, 1 or 1, 1, 1, lesions in the left main stem (≥50%) or proximal left anterior descending branch (≥70%), troponin-positive ACS, prior ischemic events (myocardial infarction (MI), ischemic stroke, peripheral arterial disease (PAD), or revascularization for CAD/PAD), diabetes mellitus (DM) managed with medication (oral hypoglycaemic agents or subcutaneous insulin therapy), or chronic kidney disease (CKD, eGFR <60 ml/min/1.73 m^2^). OPT-BIRISK bleeding criteria were as follows: ≥75 years of age, history of prior ischemic stroke, female, iron deficiency anemia (IDA). DM managed with medication (oral hypoglycaemic agents or subcutaneous insulin therapy), CKD (eGFR <60 ml/min/1.73 m^2^) exists in both criteria of bleeding and ischemic risk.

### 2.3. Follow-up and data collection

Electronic case report forms compiled by investigators and available online were used to record patient data at baseline and follow-up. Patients underwent follow-up assessment at 3, 6, 9, 12, and 24 months following enrollment by telephone, outpatient assessment, or re-hospitalization. During follow-up assessments, patients were asked about their medication status and any adverse clinical events. The routine telephone-based follow-up did not entail providing patients with any guidance regarding antiplatelet or cardiovascular treatment other than in emergencies. A clinical events committee evaluated all endpoint events.

### 2.4. Outcomes and definitions

The ischemic event at 12–24 months was the primary endpoint for this study and was defined as the composite of non-fatal MI (8), ischemic stroke, and cardiac death events. Secondary endpoints included individual components of ischemic events, all-cause mortality, and Bleeding Academic Research Consortium (BARC) (9) type 2, 3, 5 and 3, 5 bleeding at 12–24 months. Ischemic events, BARC 2, 3, 5 bleeding events, and BARC 3, 5 bleeding events at 5 years were analyzed for the external validation of the OPT-BIRISK criteria to identify bi-risk ACS patients. The protocol of OPT-CAD study recommended a standard term DAPT strategy (12 months) for patients undergoing PCI according to guideline of the time. The strategies after 12 months of antiplatelet therapy were at the discretion of the treating physician. Thus, extended DAPT was defined by treatment with both clopidogrel and aspirin at 12-month follow-up. In contrast, SAPT was defined by treatment with aspirin and clopidogrel at a 9-month follow-up but with just one antiplatelet agent at a 12-month follow-up.

### 2.5. Statistical analysis

Continuous variables are reported as means ± standard deviation (SD) and compared with Student's *t*-tests. In contrast, categorical variables are reported as frequencies (%) and were compared with chi-square or Fisher's exact tests. Univariate and multivariate logistic analyses were used to analyze the association between the OPT-BIRISK factors and ischemic and bleeding risks. Kaplan-Meier curves and log-rank tests were used to analyze time-to-even outcomes. Propensity score matching (PSM) was carried out between groups at a 1:2 ratio to compensate for potential bias among groups due to confounding factors. Variables incorporated into the model included patient characteristics such as age, sex, body mass index (BMI), DM, history of MI, history of stroke, prior PCI, tobacco use, hyperlipidemia, and PAD, as well as baseline clinical factors, including hemoglobin levels, renal function, ACS type, and ejection fraction, and procedural factors including stent diameter, total stent length, number of stents, and target vessel location. A two-sided *P* < 0.05 was the significance threshold for all analyses, and all comparisons were performed using R (v 4.1.1).

## 3. Results

### 3.1. Study population characteristics

**Figure 1:**
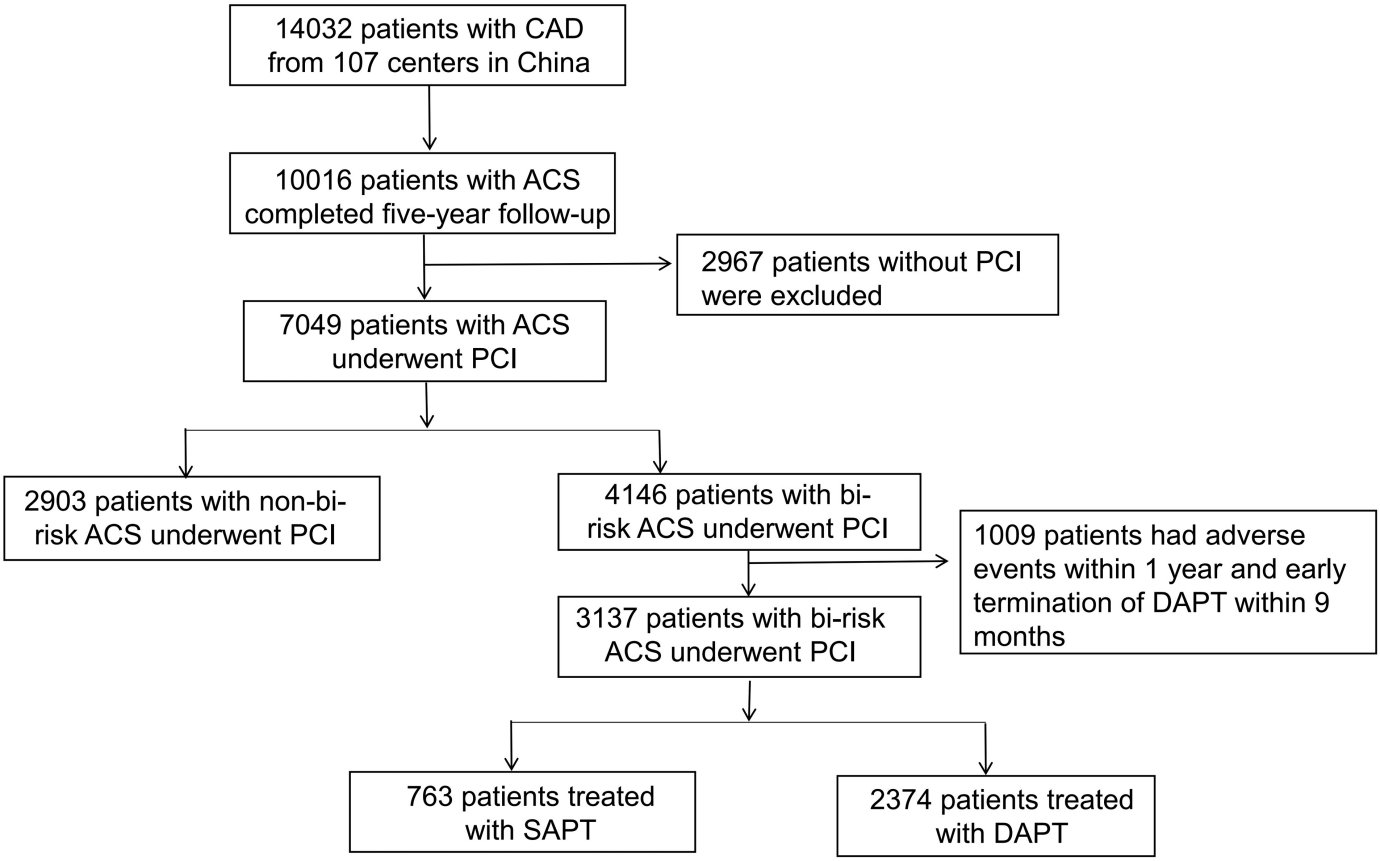
Study population flow chart.

This analysis included 7,049 ACS patients undergoing PCI, of whom 4,146 were bi-risk patients (58.8%), and 2,903 were not (41.2%). Those patients that experienced adverse events (such as MI, stroke, all-cause mortality, clinically driven revascularization, BARC type 2,3,5 bleeding, or in-stent thrombosis) within 12 months following enrollment were excluded from subsequent analyses, as were patients that underwent early DAPT termination, totaling 1,009. Of the included bi-risk patients, 2,374 (75.7%) underwent prolonged DAPT treatment, while 763 (24.3%) underwent SAPT treatment. A flowchart for patient inclusion in this study is provided in Figure 1.

### 3.2. OPT-BIRISK evaluation criteria validation

Bi-risk patients tended to be older, shows higher rates of comorbidities and previous ischemic events, and exhibit more complex coronary artery lesions than non-bi-risk patients (Supplementary Tables 1, 2). Significant differences in ischemic event frequency at 5 years were observed when comparing the bi-risk and non-bi-risk groups (11.70% vs. 5.55%, *P* < 0.001). A significantly elevated risk of BARC 2,3,5 bleeding (6.90% vs. 4.03%, *P* < 0.001) and BARC 3,5 bleeding (2.39% vs. 1.00%, *P* < 0.001) was observed among bi-risk patients relative to non-bi-risk individuals (Table 1). Consistently higher rates of other analyzed outcomes were also observed among bi-risk patients, including all-cause mortality (8.83% vs. 2.48%, *P* < 0.001), cardiac death (5.64% vs. 1.41%, *P* < 0.001), MI (3.01% vs. 2.10%, *P* = 0.018), and stroke (4.56% vs. 2.48%, *P* < 0.001) (Table 1). These results thus support the satisfactory performance of the OPT-BIRISK criteria as a means of identifying bi-risk ACS patients in this cohort. Univariate and multivariate analyses of OPT-BIRISK ischemic factors associated with ischemic events at 5 years and OPT-BIRISK bleeding factors associated with BARC 2,3,5 bleeding events are provided in Supplementary Tables 3, 4.

**Table 1:** Clinical outcomes over 5 years between bi-risk and non-bi-risk groups according to the OPT-BIRISK bi-risk evaluation criteria.

	Bi-risk (*N* = 4,146)	Non-bi-risk (*N* = 2,903)	*P*-value
Ischemic events	485 (11.70)	161 (5.55)	<0.001
Cardiac death	234 (5.64)	41 (1.41)	<0.001
MI	125 (3.01)	61 (2.10)	0.018
Stroke	189 (4.56)	72 (2.48)	<0.001
All-cause death	366 (8.83)	72 (2.48)	<0.001
BARC 2,3,5 bleeding events	286 (6.90)	117 (4.03)	<0.001
BARC 3,5 bleeding events	99 (2.39)	29 (1.00)	<0.001

Values are *n* (%). *P*-values were calculated using the log-rank test based on all available follow-up data. Ischemic event are a composite of cardiac death, myocardial infarction, or stroke. BARC indicates bleeding academic research consortium; DAPT, dual antiplatelet therapy; SAPT, single antiplatelet therapy; MI, myocardial infarction.

### 3.3. Bi-risk population distribution

Of the 3,416 enrolled bi-risk ACS patients <75 years of age that underwent PCI, the greatest number met both OPT-BIRISK ischemic criteria, after which the distribution gradually decreased with increasing ischemic risk factors. In total, 51 (1.49%), 650 (19.03%), 1,014 (29.68%), 96 (2.81%), and 805 (23.57%) patients, respectively, met 0, 1, 2, 3, or ≥4 OPT-BIRISK ischemia criteria, with a mean of 2.83 ± 1.37 ischemic criteria per patient. Patients with troponin-positive ACS (*n* = 1,466, 42.92%) met the greatest number of ischemic criteria, followed by those patients with a total stent length >30 mm (*n* = 1,272, 37.24%) (Supplementary Figures 1, 2).

The proportion of patients meeting two bleeding criteria was highest in the bi-risk group of ACS patients that underwent PCI treatment, with gradually decreasing patient distributions as the number of bleeding risk factors rose. In total, 463 (13.94%) and 2,859 (86.06%) patients, respectively, met 1 or ≥2 OPT-BIRISK bleeding criteria, with a mean of 1.44 ± 0.95 criteria per patient. Women (*n* = 1,406, 42.32%) comprised the largest number of patients meeting these bleeding criteria, followed by patients with DM managed with medications (*n* = 1,372, 41.30%) (Supplementary Figures 1, 3).

### 3.4. Effects of prolonged DAPT in bi-risk patients

Following the exclusion of 1,009 patients that experienced adverse events within 12 months and or prematurely discontinued DAPT before 9 months, 3,137 bi-risk ACS patients that underwent PCI were included in the DAPT (*n* = 2,374, 75.68%) and SAPT (*n* = 763, 24.32%) groups. When comparing these groups to assess the efficacy of prolonged DAPT treatment for 12–24 months, DAPT was found to be more commonly applied for males with lower rates of prior PCI treatment and anemia than SAPT (Table 2).

**Table 2:** Comparison of baseline clinical characteristics, procedural characteristics, and medication after discharge between patients treated with SAPT and DAPT complicated with high ischemic and bleeding risks before and after propensity score matching.

	All patients			Propensity-matched patients		
	Extended DAPT (*N* = 2,374)	SAPT (*N* = 763)	*P*-value	Extended DAPT (*N* = 1,490)	SAPT (*N* = 763)	*P*-value
Age (years)	64.82 ± 9.63	64.91 ± 9.74	0.831	64.6 ± 9.62	64.91 ± 9.74	0.477
Male	1,466 (61.75%)	433 (56.75)	0.014	859 (57.65)	433 (56.75)	0.682
BMI, kg/m^2^	24.37 ± 3.01	24.56 ± 2.92	0.128	24.59 ± 3.06	24.56 ± 2.92	0.804
Diabetes	902 (37.99%)	274 (35.91)	0.301	532 (35.7)	274 (35.91)	0.923
Hypertension	1,533 (64.57)	507 (66.45)	0.345	989 (66.38)	507 (66.45)	0.973
Hyperlipidemia	668 (28.14)	195 (25.56)	0.165	392 (26.31)	195 (25.56)	0.7
Previous MI	163 (6.87)	68 (8.91)	0.06	125 (8.39)	68 (8.91)	0.675
Previous stroke	263 (11.08)	104 (13.63)	0.056	201 (13.49)	104 (13.63)	0.927
Previous PCI	224 (9.44)	103 (13.5)	0.001	192 (12.89)	103 (13.5)	0.683
Peripheral arterial disease	31 (1.31)	7 (0.92)	0.394	10 (0.67)	7 (0.92)	0.523
Smoking history			0.734			0.984
Never	1,387 (58.42)	458 (60.03)		889 (59.66)	458 (60.03)	
Current smoker	784 (33.02)	243 (31.85)		480 (32.21)	243 (31.85)	
Ex-smoker	668 (28.14)	195 (25.56)		121 (8.12)	62 (8.13)	
Type of ACS			0.993			0.944
UA	1,328 (55.94)	425 (55.7)		819 (54.97)	425 (55.7)	
NSTEMI	319 (13.44)	103 (13.50)		203 (13.62)	103 (13.5)	
STEMI	727 (30.62)	235 (30.80)		468 (31.41)	235 (30.8)	
Anemia^a^	398 (17.03)	103 (13.62)	0.027	217 (14.56)	106 (13.89)	0.667
eGFR, mL/min/1.73 m^2^	107.14 ± 42.28	110.43 ± 39.59	0.059	110.85 ± 43.76	110.54 ± 39.66	0.871
LVEF, %	59.99 ± 8.78	60.22 ± 8.79	0.532	60.24 ± 8.49	60.22 ± 8.79	0.953
GRACE score	89.2 ± 22.52	88.33 ± 23.57	0.373	87.66 ± 22.52	88.28 ± 23.36	0.545
Lesion characteristics and procedural results						
Target lesion location						
LM	203 (4.52)	56 (3.71)	0.57	69 (4.63)	31 (4.06)	0.536
LAD	1,405 (59.18)	437 (57.27)	0.352	857 (57.52)	437 (57.27)	0.912
LCX	619 (26.07)	172 (22.54)	0.051	333 (22.35)	172 (22.54)	0.917
RCA	908 (38.25)	285 (37.35)	0.658	571 (38.32)	285 (37.35)	0.654
No. of target vessels			0.015			0.768
1	1,704 (72.05)	579 (76.59)		1,122 (75.30)	585 (76.67)	
2	533 (22.54)	152 (20.11)		315 (21.14)	153 (20.05)	
3	128 (5.41)	25 (3.31)		53 (3.56)	25 (3.28)	
Stents per patient	1.69 ± 0.93	1.66 ± 0.91	0.473	1.67 ± 0.91	1.66 ± 0.91	0.784
The total length of the stent	42.38 ± 26.86	42.57 ± 27.53	0.87	3.02 ± 0.40	3.01 ± 0.38	0.436
Average stent diameter	3.03 ± 0.39	3.01 ± 0.39	0.148	42.41 ± 26.82	42.49 ± 27.42	0.948
Medications at discharge						
Aspirin	2,348 (98.9)	758 (99.34)	0.285	1,483 (99.53)	758 (99.34)	0.554
Statins	2,313 (97.43)	733 (96.07)	0.051	1,442 (96.78)	733 (96.07)	0.383
ACEI/ARB	1,739 (73.25)	537 (70.38)	0.122	1,044 (70.07)	537 (70.38)	0.878
β-blockers	1,826 (76.92)	599 (78.51)	0.362	1,167 (78.32)	599 (78.51)	0.920
Proton pump inhibitors	925 (38.96)	311 (40.76)	0.377	606 (40.67)	311 (40.76)	0.968

Values are *n* (%) or mean ± SD. DAPT, dual antiplatelet therapy; SAPT, single antiplatelet therapy; myocardial infarction; BMI, body mass index; MI, myocardial infarction; PCI, percutaneous coronary intervention; ACS, acute coronary syndrome; UA, unstable angina; STEMI, ST-segment–elevation myocardial infarction; NSTEMI, non-ST-segment–elevation myocardial infarction; eGFR, estimated glomerular filtration rate; LVEF, left ventricular ejection fraction; DAPT, dual antiplatelet therapy; SAPT, single antiplatelet therapy; LM, left main coronary artery; LAD, left anterior descending branch; LCX, left circumflex artery; RCA, right coronary artery; ACEI/ARB, angiotensin-converting enzyme inhibitor/angiotensin II receptor blocker.

^a^ Anemia was defined as hemoglobin <13 g/dl for men or <12 g/dl for women.

**Figure 2:**
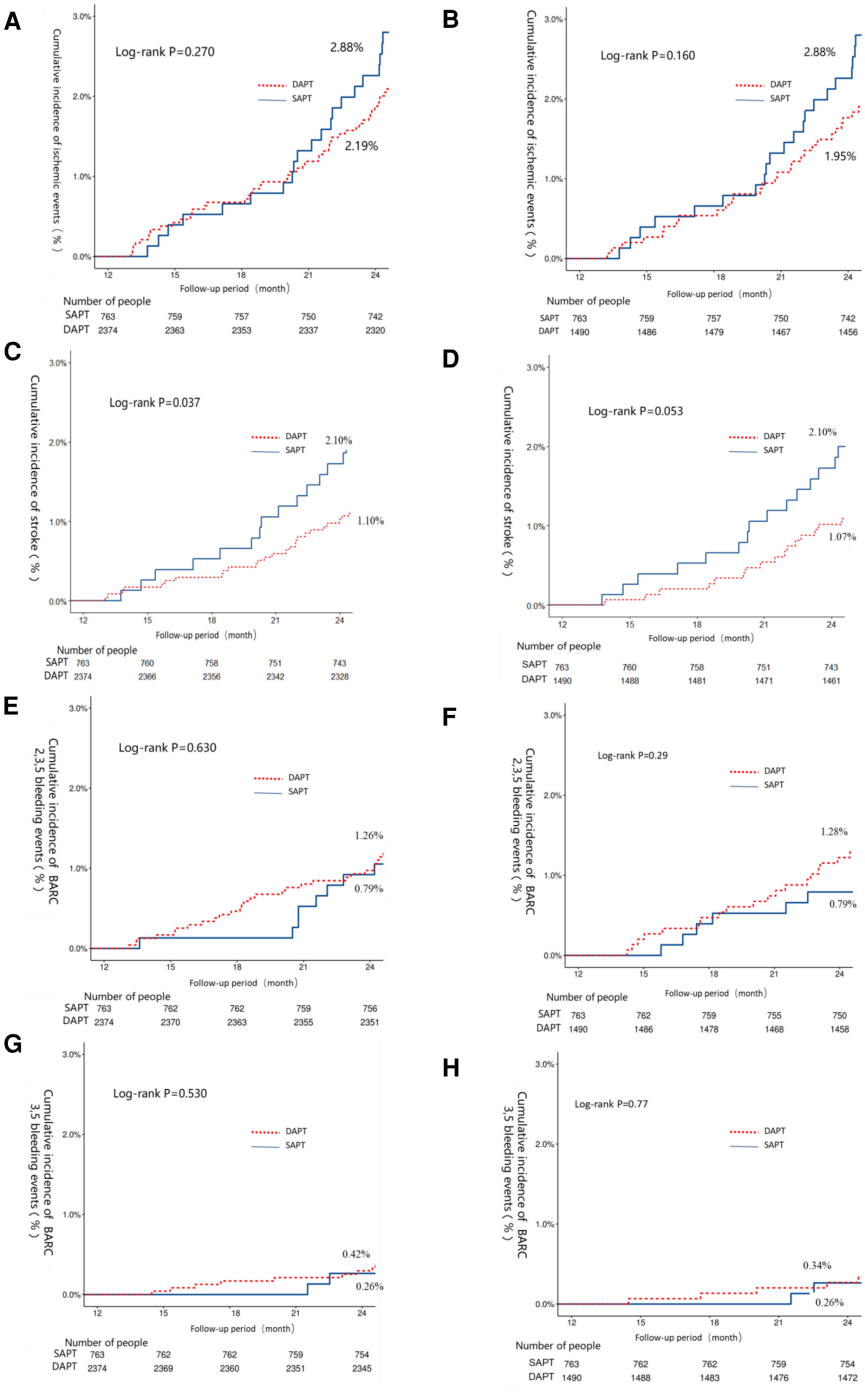
The 12–24-month Kaplan–Meier cumulative event curves for intermediate- or high-risk patients that underwent extended DAPT or SAPT treatments. Curved depict the incidence of ischemic events (**A,B**), stroke (**C,D**), BARC 2,3,5 bleeding (**E,F**), and BARC 3,5 bleeding (**G,H**). Curves are provided both before (**A,C,E,G**) and after (**B,D,F,H**) propensity score matching. Ischemic events were the primary study endpoint and were defined as the composite of myocardial infarction (MI), stroke, and cardiac death events. BARC, bleeding academic research consortium.

When comparing extended DAPT treatment to SAPT treatment, no significant differences in ischemic event incidence (2.11% vs. 2.89%, *P* = 0.273) or all-cause mortality (1.05% vs. 1.26%, *P* = 0.636, hazard ratio: 0.52, 95% CI, 0.28–0.97, number needed to treat: 91) were observed. However, DAPT-treated patients did exhibit a significantly lower risk of stroke relative to patients that underwent extended DAPT treatment (1.10% vs. 2.10%, *P* = 0.036) with similar BARC 2,3,5 bleeding risk (1.26% vs. 0.79%, *P* = 0.282) (Table 3). After matching, the extended DAPT had a lower rate of stroke (1.07% vs. 2.10%, HR: 0.51, 95% CI, 0.25–1.02, number needed to treat: 93). Ischemic and bleeding outcomes in these groups were identical before and following PSM (Table 3). Corresponding Kaplan-Meier curves are provided in Figure 2.

**Table 3:** Clinical outcomes over 24 months between patients treated with SAPT and extended DAPT complicated with high ischemic and bleeding risks before and after propensity score matching.

	All patients			Propensity-matched patients		
	Extended DAPT (*N* = 2,374)	SAPT (*N* = 763)	*P*-value	Extended DAPT (*N* = 1,490)	SAPT (*N* = 763)	*P*-value
Ischemic events	52 (2.19)	22 (2.88)	0.273	29 (1.95)	22 (2.88)	0.157
Cardiac death	17 (0.72)	4 (0.52)	0.572	8 (0.54)	4 (0.52)	1.000
MI	12 (0.51)	2 (0.26)	0.539	8 (0.54)	2 (0.26)	0.510
Stroke	26 (1.10)	16 (2.10)	0.036	16 (1.07)	16 (2.10)	0.052
All-cause death	30 (1.26)	8 (1.05)	0.636	17 (1.14)	8 (1.05)	0.843
BARC 2,3,5 bleeding events	30 (1.26)	6 (0.79)	0.282	19 (1.28)	6 (0.79)	0.295
BARC 3,5 bleeding events	10 (0.42)	2 (0.26)	0.742	5 (0.34)	2 (0.26)	1.000

Values are *n* (%). *P*-values were calculated using the log-rank test based on all available follow-up data. Ischemic event are a composite of cardiac death, myocardial infarction, or stroke. BARC indicates bleeding academic research consortium; DAPT, dual antiplatelet therapy; SAPT, single antiplatelet therapy; MI, myocardial infarction.

## 4. Discussion

This *post hoc* hypothesis-generating study conducted the first real-world evaluation of the safety and efficacy of extended DAPT treatment following PCI in ACS patients meeting the OPT-BIRISK assessment criteria. Primary study findings included the following: (1) the OPT-BIRISK criteria were able to effectively identify ACS patients facing both high risks of ischemic and bleeding events at 5 years after PCI; (2) bi-risk individuals comprise a large proportion of ACS patients following PCI, and these patients exhibit a poorer prognosis than non-bi-risk patients, including higher rates of both ischemic and bleeding events; and (3) among those bi-risk ACS patients that were able to tolerate DAPT for 12 months, the further extension of DAPT treatment was related to a significant decrease in stroke risk without any corresponding rise in bleeding events during 12–24 months.

ACS patients often present with risk factors for both ischemia and bleeding event, and certain risk factors can potentially trigger both of these adverse event types. Mohamed et al. (10) found that one in three ACS patients exhibited high-risk factors for both bleeding and ischemic events while also observing higher rates of major adverse cardiovascular events (MACE; cardiac death and reinfarction) and all-cause bleeding events as compared to non-bi-risk patients, in line with the present results. However, in the current study cohort, 58.8% of patients were classified as bi-risk individuals, since prior studies have included bi-risk patients among the overall ACS patient population. In contrast, this study specifically focused on ACS patients that had undergone PCI. In addition, this study included ACS patients ≥75 years of age, who are often excluded from other studies and are underrepresented in such research. This is noteworthy given the evidence that individuals of advanced age comprise ∼40% of patients hospitalized with ACS (11), and these individuals face a higher risk of both cardiovascular event incidence and death such that they represent the most common and clinically relevant bi-risk population. Finally, this study employed different criteria for identifying bi-risk patients relative to prior studies. In previous work, the GRACE ischemic score and the CRUSADE bleeding score were used to identify these patients even though GRACE scores only take age, cardiac enzyme levels, and laboratory findings into account without assessing comorbidities or the characteristics of coronary lesions, thereby potentially failing to detect bi-risk patients.

Assessing ischemic and bleeding risks in individual patients receiving antiplatelet therapy is crucial to balance the potential benefits and harm. Numerous randomized trials have investigated the optimal antiplatelet regimen in various patient cohorts (3, 12–17). Strategies developed to mitigate the risk of bleeding, including shortening DAPT duration, P2Y12 inhibitor monotherapy, and de-escalation, may prevent the exposure to an excessive bleeding hazard in patients deemed at high bleeding risk upfront. In turn, patients at non-high bleeding risk might consider a standard or prolonging DAPT strategy if tolerated. Honestly, there is no doubt that patients at increased ischemic risk could benefit from more intensive antiplatelet therapy, while those at increased bleeding risk warrant less intensive therapy. However, in real-world practice, many patients are at increased risk for ischemic and bleeding events. In the present study, we found that about 60% of patients possessed high bleeding and ischemic risk characteristics. Compared with the absence of relevant features, the patients at high risk of both ischemic and bleeding events had a 2-fold increased risk of ischemic events, 3.5-fold increased risk of all-cause mortality, and 2-fold increased risk of major bleeding. Howbeit, the optimal antithrombotic treatment regimen in this patient subset remains unclear. In the DAPT study, the clinical benefit (MACCE: 4.3% vs. 5.9%) of extended thienopyridine treatment was tempered by increased bleeding events (BARC types 2, 3, or 5 bleeding: 5.6% vs. 2.9%) (3). The current study detected that extended DAPT strategy was associated with a slight risk reduction of ischemic events (1.95% vs. 2.88%), especially stroke (1.07% vs. 2.10%), without excessive risk of major bleeding complications (0.34% vs. 0.26%). The inconsistent results and lower incidence of bleeding and ischemic events might contribute to a high proportion of bleeding and ischemic events, which might contribute to a high proportion of the trial population treated with prasugrel, ethnic differences, procedural improvements, and changes in bleeding avoidance strategies over time. Nevertheless, the incidence of ischemic and bleeding events in the present study was similar to that observed between 1 and 2 years after PCI in other trials involving East Asian populations (18, 19). Considering the contemporary clinical practice, extended P2Y12 inhibitor monotherapy with clopidogrel might be an optimal antiplatelet strategy. A multicenter, prospective, double-blinded, active-controlled randomized trial, OPT-BIRISK, is designed to examine the efficacy and safety of extended P2Y12 inhibitor monotherapy with clopidogrel in patients at high risk for both ischemic and bleeding complications who had completed 9–12 months of DAPT after drug-eluting stent implantation, which will establish the optimal intensity of antiplatelet therapy (4).

Moreover, the applicability and robustness of assessment algorithm is extremely important. Briefly, the OPT-BIRISK criteria compose of twelve clinical high ischemic criteria (binary classification, “Yes” or “No”) and six high bleeding criteria (binary classification, “Yes” or “No”). Not like GRACE (20) and PRECISE-DAPT score (21), the patients could be evaluated simply and quickly. Nevertheless, an app or plug-in component, which could capture all factors relating to the OPT-BIRISK criteria in the electronic medical records, evaluate them automatically, and then quickly preset the ischemic and bleeding risk of patients, is convenient to use for clinicians and to validate in other population.

## 5. Limitation

There are several limitations to this study. First, this was an observational study in China and individual DAPT regimens based on individual patient conditions existed such that not all patients may have used their medications in strict accordance with the defined DAPT groupings. As these analyses were conducted by classifying those patients remaining adherent to a DAPT regimen at 12 months postoperatively as exhibiting a prolonged (>12-month) DAPT treatment duration with no consideration for discontinuation after 12 months, the benefits of extended DAPT treatment to the risk of bleeding or ischemic events are likely underestimated. Moreover, the present trial was conducted solely in a Chinese population. Additional randomised trials are necessary to investigate the optimal antiplatelet strategy after a mandatory DAPT period further in a Western population of high-risk ACS patients. Secondly, despite the present study was adjusted by propensity score matching, we were unable to evaluate and control some unmeasured covariates which might influence the choice of antiplatelet therapy, such as physician characteristics. Additionally, owing to the observational nature of the study, the results could only provide the evidence of association. Third, considering clopidogrel was the only P2Y12 inhibitor in the present study, which might limited the promotion value in the era of potent P2Y12 inhibitors. However, recent observational studies showed that about 57%–76% of ACS patients undergoing PCI received clopidogrel-based DAPT strategy at discharge (22, 23), reflecting the gap the between guidelines-indicated and the clinical practice. For clinicians, it's important to make clinical judgments and consequently tailor the intensity and the duration of antiplatelet therapy to individual patients according to the ischemic and bleeding risk. At last, owing to only ∼3% patients received clopidogrel monotherapy in the SAPT group, analyses of the utilized type of monotherapy or the effects of different forms of medication on patient prognosis could not be conducted.

## 6. Conclusion

The OPT-BIRISK criteria may assist clinicians in identifying ACS patients at risk of recurrent ischemic and bleeding complications following PCI. An extended DAPT strategy, compared to antiplatelet monotherapy, could offer prospective benefits in reducing the risk of stroke without a substantial increase in major bleeding problems among patients who remained event-free after 12 months of dual antiplatelet therapy (DAPT). However, these interesting results must be validated in larger, randomized investigations.

## Data Availability

The raw data supporting the conclusions of this article will be made available by the authors, without undue reservation.
