# Supplementary material for: Effect of dual antiplatelet therapy prolongation in acute coronary syndrome patients with both high ischemic and bleeding risk: insight from the OPT-CAD study
Source: Front Cardiovasc Med. 2023 Sep 8;10:1201091. doi: 10.3389/fcvm.2023.1201091 (PMC10514577; doi:10.3389/fcvm.2023.1201091)

**SUPPLEMENTAL MATERIAL**

**CONTENT**

1. Supplemental Methods: None
2. Supplemental Tables: 5
3. Supplemental Figures and Figure Legends: 3
4. Supplemental References: None

**Supplemental Table 1**. Baseline clinical characteristics and medication after discharge in the bi-risk and non-bi-risk groups according to the OPT-BIRISK bi-risk evaluation criteria.

|  | Bi-risk (N = 4146) | Non-bi-risk (N = 2903) | P-value |
| --- | --- | --- | --- |
| Age (years) | 65.18±9.65 | 53.36±8.04 | <0.001 |
| Male | 2501(60.32) | 2818(97.07) | <0.001 |
| BMI, kg/m^2^ | 24.4±3.00 | 24.86±2.89 | <0.001 |
| Diabetes | 1547(37.31) | 157(5.41) | <0.001 |
| Hypertension | 2725(65.73) | 1413(48.67) | <0.001 |
| Hyperlipidemia | 1116(26.92) | 876(30.18) | 0.003 |
| Previous MI | 310(7.48) | 179(6.17) | 0.033 |
| Previous stroke | 502(12.11) | 0(0) | <0.001 |
| Previous PCI | 442(10.66) | 258(8.89) | 0.026 |
| Peripheral arterial disease | 59(1.42) | 11(0.38) | <0.001 |
| Smoking history |  |  | <0.001 |
| Never | 2422(58.42) | 879(30.28) |  |
| Current smoker | 1359(32.78) | 1792(61.73) |  |
| Ex-smoker | 365(8.80) | 232(7.99) |  |
| Type of ACS |  |  | <0.001 |
| UA | 2286(55.14) | 1512(52.08) |  |
| NSTEMI | 576(13.89) | 316(10.89) |  |
| STEMI | 1284(30.97) | 1075(37.03) |  |
| Anemia^a^ | 683(16.68) | 11(0.38) | <0.001 |
| eGFR, mL/min/1.73 m^2^ | 107.39±41.69 | 120.78±35.58 | <0.001 |
| LVEF, % | 59.87±8.93 | 60.8±8.40 | <0.001 |
| GRACE score | 89.80±22.99 | 68.90±18.95 | <0.001 |
| Medications at discharge |  |  |  |
| Aspirin | 4079(98.38) | 2877(99.10) | 0.009 |
| Statins | 4017(96.89) | 2799(96.42) | 0.276 |
| ACEI/ARB | 3010(72.60) | 2016(69.45) | 0.004 |
| β-Blockers | 3199(77.16) | 2198(75.71) | 0.159 |
| Proton pump inhibitors | 1645(39.68) | 1088(37.48) | 0.062 |
| β-Blockers | 3199(77.16) | 2198(75.71) | 0.159 |
| Proton pump inhibitors | 1645(39.68) | 1088(37.48) | 0.062 |

Note: Values are n (%) or mean ± SD

Abbreviations: MI, myocardial infarction; PCI, percutaneous coronary intervention; ACS, acute coronary syndrome; UA, unstable angina; STEMI, ST-segment–elevation myocardial infarction; NSTEMI, non–ST-segment–elevation myocardial infarction; eGFR, estimated glomerular filtration rate; LVEF, left ventricular ejection fraction; ACEI/ARB, angiotensin converting enzyme inhibitor/angiotensin II receptor blocker.

a. Anemia was defined as hemoglobin <13 g/dL for men or <12 g/dL for women.

**Supplemental Table 2**. Comparison of lesion characteristics and procedural results between bi-risk and non-bi-risk groups according to the OPT-BIRISK bi-risk evaluation criteria.

|  | Bi-risk (N = 4146) | Non-bi-risk (N = 2903) | P-value |
| --- | --- | --- | --- |
| Target lesion location |  |  |  |
| LM | 186(4.49) | 88(3.03) | 0.002 |
| LAD | 2386(57.55) | 1661(57.22) | 0.781 |
| LCX | 1037(25.01) | 695(23.94) | 0.304 |
| RCA | 1568(37.82) | 933(32.14) | <0.001 |
| No. of target vessels |  |  | <0.001 |
| 1 | 3042(73.91) | 2336(81.14) |  |
| 2 | 857(20.82) | 447(15.53) |  |
| 3 | 217(5.27) | 96(3.33) |  |
| Stents per patient | 1.69±0.93 | 1.53±0.85 | <0.001 |
| Total length of stent | 42.79±26.9 | 38.66±24.68 | <0.001 |
| Average stent diameter | 3.02±0.39 | 3.11±0.42 | <0.001 |

Note: Values are n (%) or mean ± SD.

Abbreviations: LM, [left](javascript:;) [main](javascript:;) [coronary](javascript:;) [artery](javascript:;); LAD, [left](javascript:;) [anterior](javascript:;) [descending](javascript:;) [branch](javascript:;); LCX, [left](javascript:;) [circumflex](javascript:;) [artery](javascript:;); RCA, [right](javascript:;) [coronary](javascript:;) [artery](javascript:;).

**Supplemental Table 3.** Univariate and multivariate analysis of OPT-BIRISK ischemic factors for ischemic events at 5 years.

| Characteristics | Univariate OR (95%CI) | | Multivariate OR (95%CI) | |
| --- | --- | --- | --- | --- |
|  | OR (95%CI) | *P* value | OR (95%CI) | *P* value |
| Age ≥75 y old | 2.57(2.12-3.12) | <0.001 | 1.10(1.07-1.12) | <0.001 |
| Multivessel coronary artery disease | 0.87(0.71-1.06) | 0.161 | 0.98(0.96-1.00) | 0.030 |
| Target lesion requiring total stent length≥30 mm | 1.13(0.97-1.31) | 0.122 |  |  |
| Thrombotic target lesion | 1.11(0.88-1.40) | 0.380 |  |  |
| Bifurcation lesion | 0.76(0.51-1.13) | 0.176 |  |  |
| Left main (≥50%) or proximal LAD (≥70%) lesion | 0.92 (0.79-1.07) | 0.257 |  |  |
| Troponin-positive ACS | 1.29 (1.11-1.50) | 0.001 | 1.03(1.01-1.04) | 0.001 |
| Previous ischemic events | 2.19 (1.84-2.62) | <0.001 | 1.09(1.07-1.12) | <0.001 |
| Diabetes mellitus | 1.50(1.26-1.77) | <0.001 | 1.04(1.02-1.06) | <0.001 |
| Chronic kidney disease | 2.02(1.69-2.43) | <0.001 | 1.05(1.03-1.08) | <0.001 |

MI, myocardial infarction; LAD, left anterior descending; PAD, peripheral artery disease; CAD, coronary artery disease; CI, confidence interval; OR, odds ratio; Anemia was defined as hemoglobin <13 g/dL for men or <12 g/dL for women.

**Supplemental Table 4.** Univariate and multivariate analysis of OPT-BIRISK bleeding factors for BARC 2,3,5 bleeding events at 5 years.

| Characteristics | Univariate OR (95%CI) | | Multivariate OR (95%CI) | |
| --- | --- | --- | --- | --- |
|  | OR (95%CI) | *P* value | OR (95%CI) | *P* value |
| Age ≥75 y old | 1.80(1.37-2.37) | <0.001 | 1.04(1.02-1.05) | <0.001 |
| Female | 1.12(0.89-1.41) | 0.335 |  |  |
| Anemia | 1.65(1.24-2.19) | 0.001 | 1.03(1.01-1.05) | 0.005 |
| Previous stroke | 1.39(0.98-1.96) | 0.065 | 1.02(1.00-1.04) | 0.083 |
| Diabetes mellitus | 1.17(0.92-1.49) | 0.194 |  |  |
| Chronic kidney disease | 1.24(0.94-1.62) | 0.126 |  |  |

CI, confidence interval; OR, odds ratio; Chronic kidney disease defined as an estimated glomerular filtration rate <60 mL/min per 1.73 m^2^ or creatinine clearance <60 mL/min.

**Supplemental Table 5**. Clinical outcomes over 12-24 months between patients treated with aspirin monotherapy, clopidogrel monotherapy and extended DAPT.

|  | Aspirin monotherapy  (N=738) | Clopidogrel monotherapy  (N=25) | Extended DAPT  (N =2374) | P value |
| --- | --- | --- | --- | --- |
| Ischemic events | 20 (2.71%) | 2 (8.00%) | 52 (2.19%) | 0.126 |
| Cardiac death | 3 (0.41%) | 1 (4.00%) | 17 (0.72%) | 0.082 |
| MI | 2 (0.27%) | 0 (0.00%) | 12 (0.51%) | 0.667 |
| Stroke | 15 (2.03%) | 1 (4.00%) | 26 (1.10%) | 0.078 |
| All-cause death | 7 (0.95%) | 1 (4.00%) | 30 (1.26%) | 0.349 |
| BARC types 2,3,5 bleeding | 6 (0.81%) | 0 (0.00%) | 30 (1.26%) | 0.522 |
| BARC types 3,5 bleeding | 2 (0.27%) | 0 (0.00%) | 10 (0.42%) | 0.807 |

Note: Values are n (%). P-values were calculated using the log-rank test based on all available follow-up data. Ischemic event are a composite of cardiac death, myocardial infarction, or stroke. BARC indicates bleeding academic research consortium; DAPT, dual antiplatelet therapy; SAPT, single antiplatelet therapy; MI, myocardial infarction.

**Supplemental Figure 1**. Distribution of OPT-BIRISK criteria.


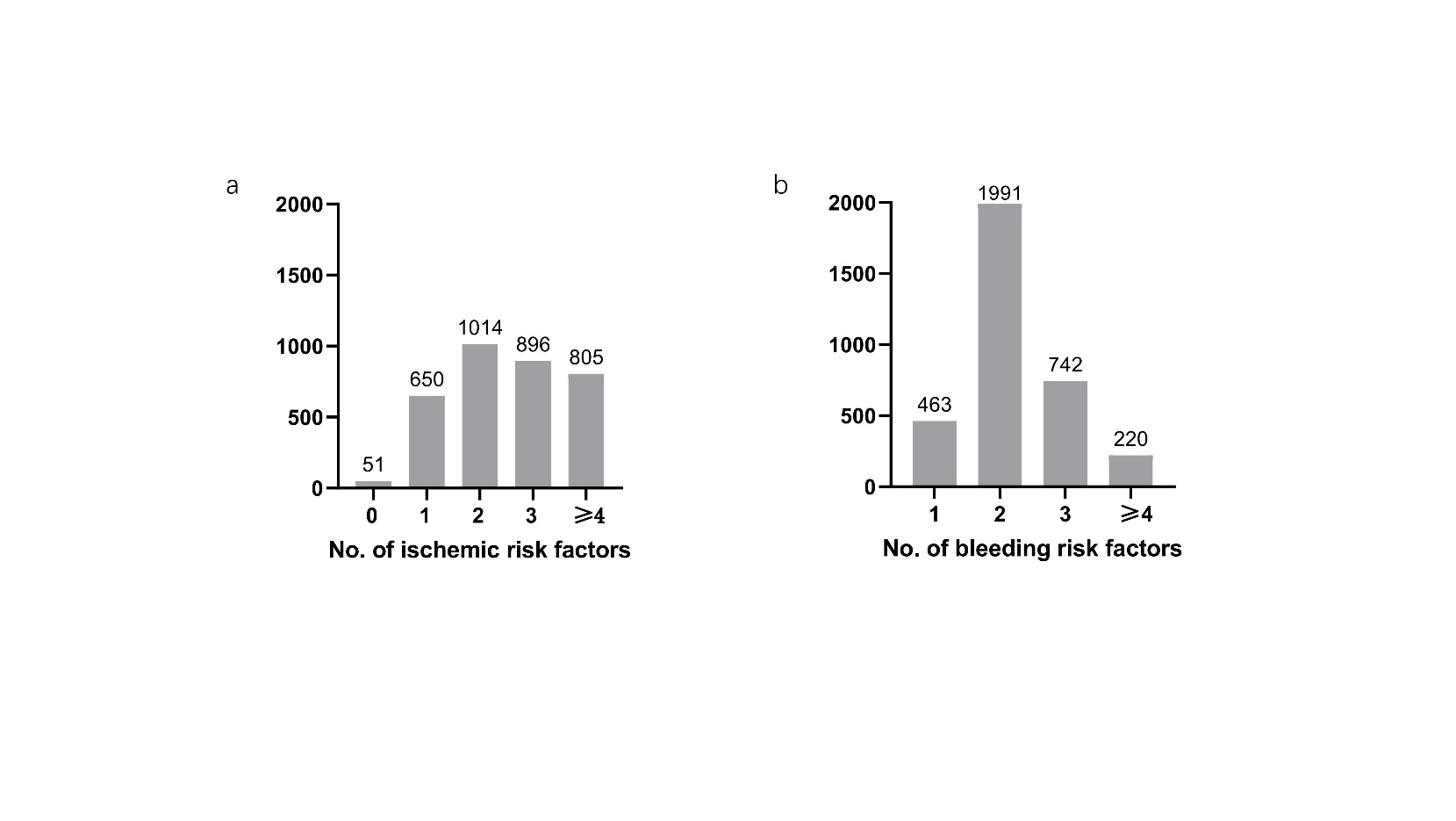


**Supplemental Figure 2**. Distribution of OPT-BIRISK ischemic criteria.


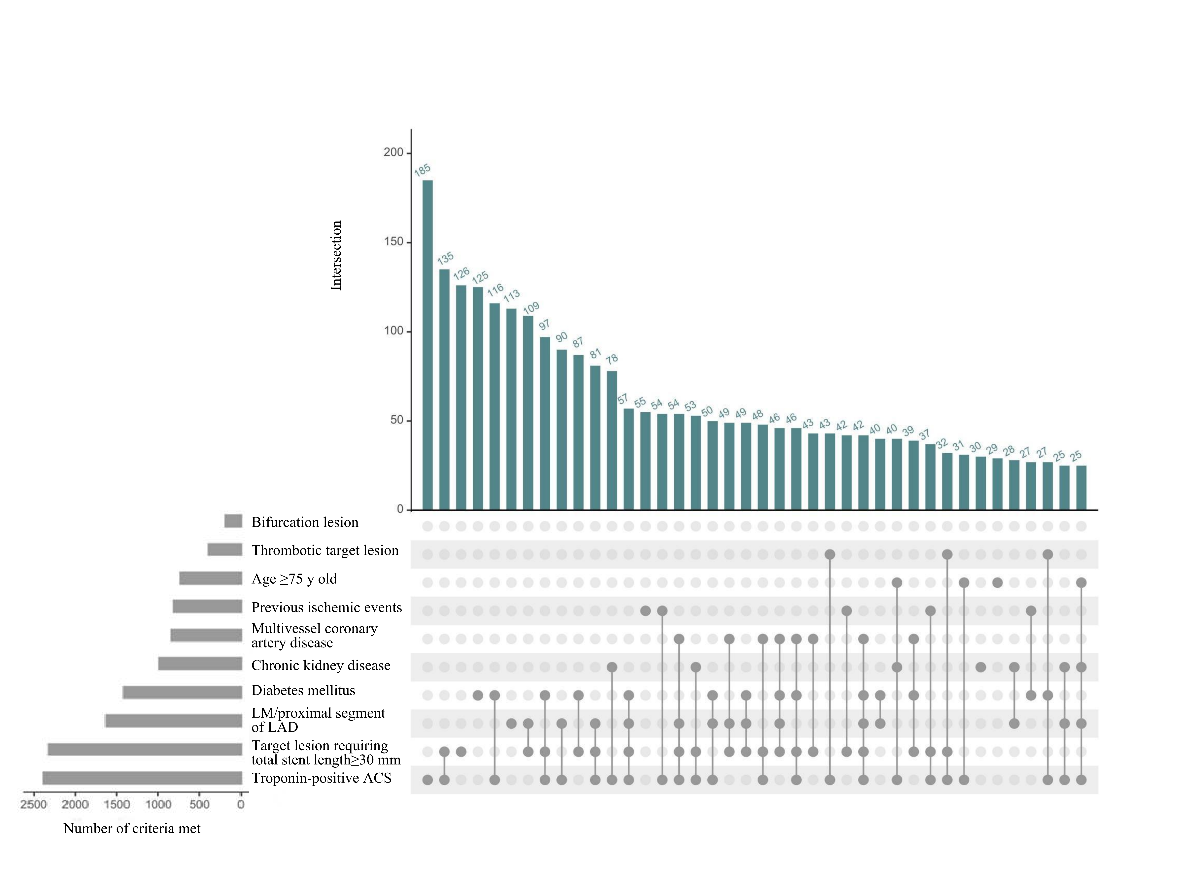


**Supplemental Figure 3**. Distribution of OPT-BIRISK bleeding criteria.


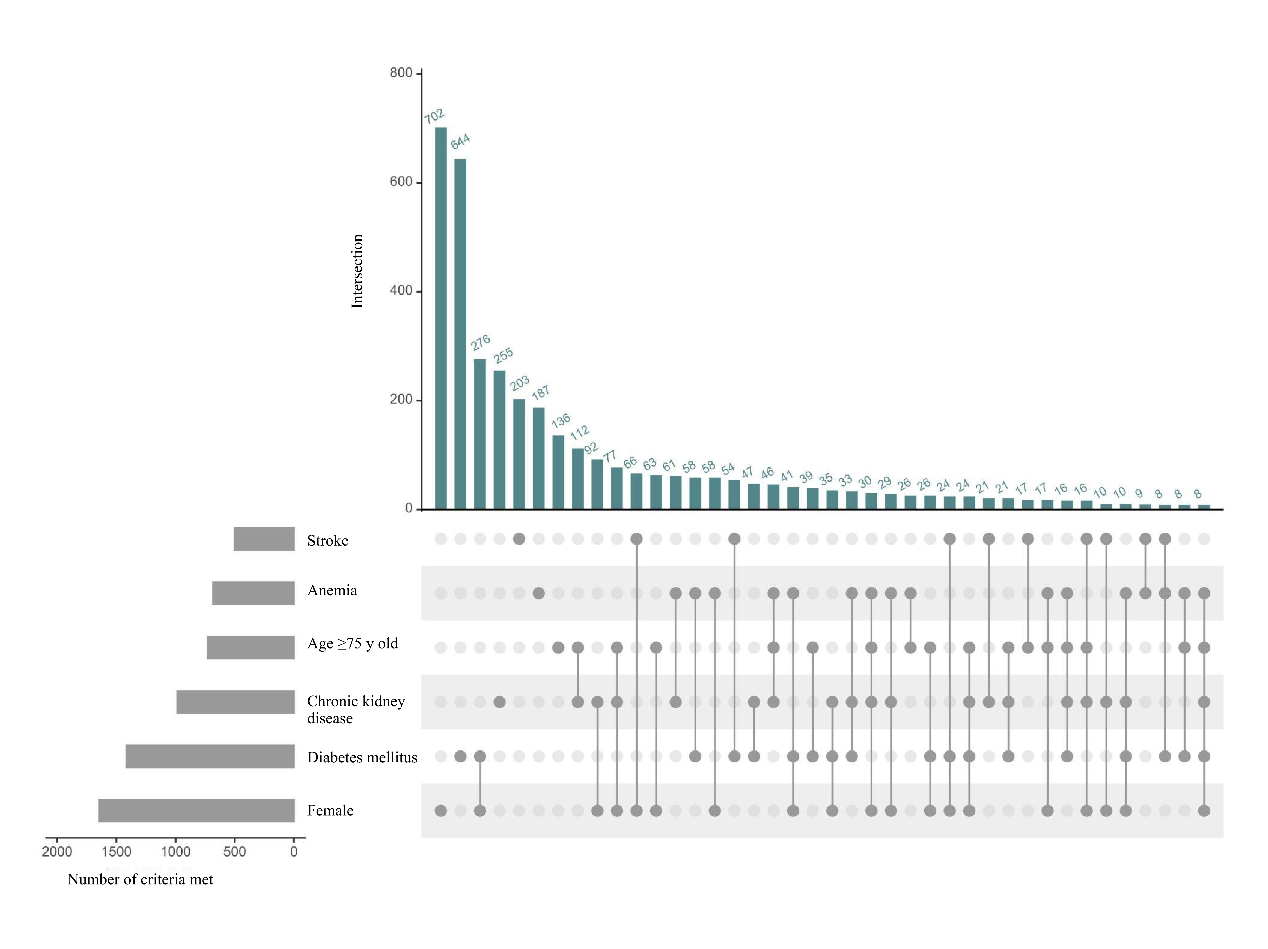

Supplement: Supplementary file 1 [file Table1.docx]
